# Force Production and Coordination from Older Women in Water Fitness Exercises

**DOI:** 10.3390/healthcare9081054

**Published:** 2021-08-16

**Authors:** Catarina C. Santos, Daniel A. Marinho, Luís B. Faíl, Henrique P. Neiva, Mário J. Costa

**Affiliations:** 1 Department of Sport Sciences, University of Beira Interior, 6201-001 Covilhã, Portugal; dmarinho@ubi.pt (D.A.M.); luis.brandao.fail@ubi.pt (L.B.F.); hpn@ubi.pt (H.P.N.); 2 Department of Sports Sciences, Polytechnic Institute of Guarda, 6300-559 Guarda, Portugal; mario.costa@ipg.pt; 3 Research Center in Sports Sciences, Health Sciences and Human Development, CIDESD, 5001-801 Vila Real, Portugal

**Keywords:** propulsive force, asymmetries, motor control, older women, cadence, aquatic exercise

## Abstract

The aim of this study was to compare bilateral propulsive forces and coordination while exercising at static and dynamic conditions in the water. A total of 27 older women (age: 65.1 ± 6.7 years old) performed the following exercises: (i) horizontal upper-limbs adduction (HA; static condition) and (ii) rocking horse (RH; dynamic condition) through an incremental protocol with music cadences from 105 up to 150 b·min^−1^. The duration of each trial was set at 30 second (sec). Propulsive peak force (in Newton, N) of dominant (PF_D_) and nondominant (PF_ND_) upper limbs was retrieved using hand sensors coupled to a differential pressure system. Significant differences in force production were found between static and dynamic exercises at higher cadences (120, 135, and 150 b·min^−1^). The static condition elicited higher bilateral propulsive forces and a more symmetric pattern. The in-water static exercise with bilateral action from the upper limbs proved to be the most appropriate strategy for older women to work strength and to reduce asymmetries.

## 1. Introduction

Water exercise has been widely recommended to enhance the quality of life and the health-related parameters [1,2,3]. Taking into account the diversity of water programs, we may find participants from different age groups, fitness levels, and genders. Still, the presence of older adults is more frequent, mostly from the women cohort.

Older women’s motivations to exercise in water are diverse, but mainly related to gain health benefits [4]. The maintenance of the physiological capacity [5] and/or improvement in body composition [2] is desirable. The improvement in muscle strength is also a key factor [6]. It is clear that changes in motor control are expected with advancing age [7], which may affect coordination [8]. Furthermore, people above 60 years old are expected to experience a decline in the neuromuscular system [9], affection force production. However, there is a gap in the literature on how older women respond (i.e., force production) while performing different water fitness exercises.

To date, most studies evaluating force production during water fitness programs or after a single bout of exercise used land-based setups [10]. Regular strength assessments about the effects of water programs were conducted using gym-workout [11] or isokinetic [12] land-based machines. However, some progress was made in the past years by the development of a more friendly user apparatus to evaluate the capability to apply force on the water. Differential pressure sensors were created to allow displacing the body through the water without any constraints [13]. Those sensors measure the water pressure differences between the palmar–plantar surface (low-pressure field) and dorsal surface (high-pressure field) during an unsteady motion [13,14,15]. To date, a few studies used pressure sensors to measured propulsive forces during water exercises. In fact, those studies recruited younger adults as subjects [16] or compared alternated with simultaneous actions in standing positions [17]. At least for young adults (21.23 ± 1.51 years old), Santos et al. [18] noted that the musical cadence of 135 beats per minute (b·min^−1^) seems to be appropriate to maintain the symmetric motion. However, the manner in which older respond to different modes of exercise (static vs. dynamic), as well as how they adapt their coordination while increasing intensity, still remains to be answered.

We have here a chance to clarify how older women respond to different modes of exercise in the water compelling bilateral actions. This will help practitioners to choose the most appropriate exercise and set the more comfortable music cadence for older women in order to achieve desirable coordination and avoid potential long-term injuries.

The aim of this study was to compare bilateral propulsive forces and coordination while exercising at static and dynamic conditions in the water. It was hypothesized that the static exercise mode would provide a more desirable exertion regarding force production and symmetry.

## 2. Materials and Methods

### 2.1. Participants

The sample size required was computed beforehand (GPower, v.3.1.9, University of Kiel, Germany). Thus, 27 older women (age: 65.1 ± 6.7 years old; body mass: 70.9 ± 9.6 kg; height: 153.2 ± 31.4 cm; body mass index: 27.9 ± 3.5 kg/m^2^) participated in this study. The inclusion criteria were defined as follows: (i) having ≥ 60 years old; (ii) clinically healthy at the beginning of the study; (iii) physically active, with at least one year of experience in water fitness programs; (iv) not having any history of musculoskeletal or neurologic injury, conditions, or syndromes diagnosed in the past six months. All women were informed of the benefits and experimental risks prior to signing an informed consent document.

### 2.2. Design and Procedures

**Figure 1:**
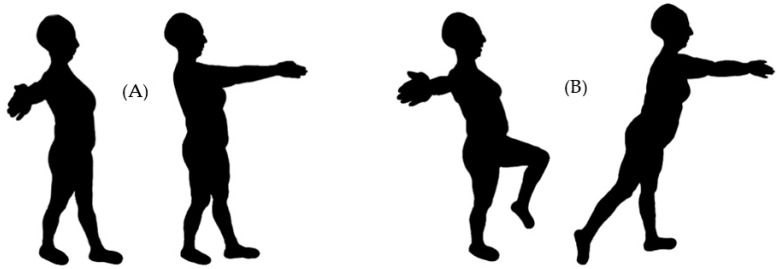
The static and dynamic water fitness exercises: “horizontal adduction” (**A**) and “rocking horse” (**B**).

A 25 m indoor pool (12.5 m width and maximal depth of 1.80 m) with mean water and air temperature of 29.5 °C and 31 °C, respectively, and relative humidity of 65% was considered for the randomized crossover study. Women were assigned to perform in two different days, separated within one week, and at the same time of the day (morning), two water fitness exercises with different biomechanical strategies (Figure 1): (i) horizontal upper-limbs adduction (HA; static condition) and (ii) rocking horse with horizontal upper-limbs adduction (RH; dynamic condition). The description of each water fitness is reported elsewhere [16] and the level of the water surface was established at the near xiphoid process [19]. Since participants presented different heights, the water surface boundary for each participant was modified and controlled by the water depth of the pool. The participants performed a 3 minutes (min) warm-up before the assessments, as reported elsewhere [17].

An incremental protocol with four music cadences (105, 120, 135, and 150 b·min^−1^) was considered for each exercise. The music cadences increased by 15 b·min^−1^ every 30 second (sec). Both exercises were performed at “water tempo” [20], which allows the synchronization with the specific movement, and the music cadence was controlled by a metronome (Korg, MA-30, Tokyo, Japan) plugged into a sound system. Verbal and visual cues were given by an expert water fitness instructor. The test ended when [16,17] (i) the participant decreased the amplitude of the movement, (ii) failed to maintain the music cadence, or (iii) finalized the 30 s trial.

### 2.3. Measures

Propulsive forces were assessed using a hand differential pressure system (Aquanex System, Swimming Technology Research, Richmond, VA, USA) with a 0.2% measurement error [21]. The system is composed of two pressure sensors (type A, Swimming Technology Research, Richmond, VA, USA) that were positioned between the third and fourth metacarpals to measure the pressure between the palmar and dorsal surfaces of both hands. They allowed assessing the peak force of dominant (PF_D_) and nondominant (PF_ND_) upper limbs in Newton (N). A signal-processor (AcqKnowledge v.3.7.3, Biopac Systems, Santa Barbara, CA, USA) was used to export data with a 5 Hz cutoff low-pass 4th order Butterworth filter upon residual analysis. The first positive and negative peaks (one cycle) were discarded, being considered the subsequent 5 cycles. The higher value (positive) was retrieved for further analysis. Symmetry index (SI, %), as a coordination measure, was estimated as proposed by Robinson et al. [22].

(1) $$\mathrm{SI}\;\left(\%\right)=\frac{2\left({\mathrm{PF}}_{D}-{\mathrm{PF}}_{\mathrm{ND}}\right)}{\left({\mathrm{PF}}_{D}+{\mathrm{PF}}_{\mathrm{ND}}\right)}\times 100$$

### 2.4. Statistical Procedures

Exploratory data analysis was used to identify potential outliers. The Shapiro–Wilk was used to confirm the normality of the distributions (*p* > 0.05). Data were expressed as mean and standard deviation (SD). Student’s *t*-test was conducted to compare all dependent variables. Repeated-measures ANOVA, followed by the Bonferroni post hoc test, was used to verify differences in bilateral propulsive force between music cadences. The symmetry data were interpreted as follows [22]: if SI = 0%, perfect symmetry; if 0% > SI < 10%, symmetric motion; if SI ≥ 10%, asymmetric motion. The effect size (ES) was computed based on Cohen’s *d* [23] and interpreted according to author’s recommendation: (i) small (0.20 ≤ *d* < 0.50); (ii) moderate (0.50 ≥ *d* < 0.80); (iii) large (*d* ≥ 0.80). The level of statistical significance was set at *p* ≤ 0.05.

## 3. Results

Table 1 shows the propulsive peak force for dominant (PF_D_) and nondominant upper-limb (PF_ND_). Values seem to increase from slower to faster cadences, in the two exercises and both upper limbs. Significant differences between exercises were observed for PF_D_ and PF_ND_ at a cadence of 120 and 150 b·min^−1^, and for PF_ND_ at 135 b·min^−1^. A large ES was found for PF_ND_ at a cadence of 120 b·min^−1^.

The comparison between upper limbs at the same exercise, and music cadence is also shown in Table 1. Significant differences were found between PF_D_ and PF_ND_ during the static condition at cadence of 105 (*p* < 0.01; *d* = 0.52) and 135 b·min^−1^ (*p* = 0.05; *d* = 0.40), whereas the dynamic condition showed at cadence of 120 (*p* < 0.01; *d* = 0.52), 135 (*p* < 0.01; *d* = 0.62) and 150 b·min^−1^ (*p* = 0.02; *d* = 0.62).

**Figure 2:**
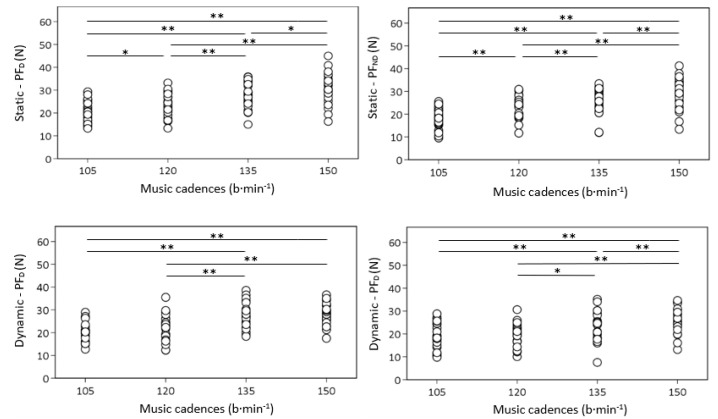
Comparison of music cadences according to the PF_D_ and PF_ND_ for two water fitness exercises. * *p* ≤ 0.05; **, *p* ≤ 0.01.

Figure 2 depicts the comparison between music cadences in PF_D_ and PF_ND_ for the two water fitness exercises. Significant differences were found between overall music cadences for PF_D_ while exercising the static condition. The dynamic condition showed differences between most of the music cadences for both limbs. No differences were found between cadence 105–120 b·min^−1^ for PF_D_ and PF_ND_, and cadence 135–150 b·min^−1^ for PF_D_ during the dynamic condition.

The symmetry index (SI) for both exercises was above 10% (cutoff value) across the incremental protocol (Table 2). No differences were found between exercises at the same music cadence. Nevertheless, cadence of 105 b·min^−1^ showed a value near to significance (*p* = 0.06, *d* = 0.51).

## 4. Discussion

This study aimed to analyze and compare bilateral propulsive force and coordination throughout an incremental protocol between two water fitness exercises. The main findings were that the bilateral propulsive force increased throughout an incremental protocol showing differences between the static and dynamic conditions mostly at a higher intensity. Both exercises elicited an asymmetrical pattern but with smaller values for the static condition.

Older women were capable to produce propulsive forces between ≈18 N (105 b·min^−1^) to ≈31 N (150 b·min^−1^) in both exercises. This is lower than the values of PF_D_ near 50 N (150 b·min^−1^), previously reported for young women and men at the same exercises [16]. The in-nature process can explain differences between age groups. In addition, at some point, inter-subject variability can be increased even when responding to the same mode of exercise. Aging is associated with a decline in skeletal mass [24], muscle strength [25], and explosive force production [26]. Fast-twitch muscle fibers decrease, as well as the motor units [27,28], linked to a progressive loss of alpha motoneurons [29]. Alterations in muscle function increase variability in force control [30], affecting the ability to perform certain motor tasks [31]. Thus, water fitness instructors should pay attention to heterogeneous age groups and develop strength properly.

There was a trend to see different propulsive force values when comparing both exercises. Here, the static condition showed a trend to present higher values for both limbs. The ability to remain in an upright stance position starts to become a challenge for older adults [32]. It is well documented that motor control and balance declines with aging [7,33], leading to an increase in the risk of falls [34]. Probably, the participants experienced a more difficult motion pattern by adding movement from the remaining parts of the body (e.g., lower limbs). Exercises that involve movement at multiple joints are susceptible to a bilateral deficit on maximum strength [35]. Moreover, dual tasks require a higher demand for processing the information [36,37]. This explains the lower force values found on dynamic condition since requires higher cognitive processing to perform the upper and lower limbs simultaneously. Meanwhile, the multiple hops may create instability and, consequently, lead to a force production decrease in this more complex condition.

Differences in propulsive forces were found between most of the music cadences in both static and dynamic conditions. In addition, the differences between dominant and nondominant limbs were found at higher cadences. At least one study reported increases in propulsive forces in young participants through an incremental protocol [16]. This seems to be an expected behavior and not an age-related factor. The cadence effect was already observed in other kinds of domains such as physiologic response [38], muscle activity [39], kinematics [40], and ground reaction forces [41] at various exercises or extensions. Although it is clear that force output increases with cadence, it remains undefined which is the optimal music cadence to work strength in this group of subjects. This should be clarified taking into account both force and symmetry outputs.

Although no differences were found between both conditions at the same cadence, the static condition elicited a more symmetric pattern. Interestingly, young adults showed a similar pattern while performing a static and dynamic condition [18]. Understanding the force-generating for assessing the inter-limb symmetry leads to a clear understanding of injury predisposition [42]. For instance, coordination can be affected by neuromuscular fatigue [43] and muscular imbalances [44]. Our results showed that none of the music cadences promoted a symmetric motion. Although the cadence of 150 b·min^−1^ elicited higher bilateral propulsive forces for both conditions, it seems that the static condition at lower music cadences is more suitable to reduce asymmetries for this population. Water fitness instructors should be aware of the correct use of music cadence and different types of exercises/variants to reduce hypothetical injuries and to build strength correctly.

The following limitations of the present research can be indicated: (i) not including a kinematic analysis to control the range of motion; (ii) not using a more heterogeneous sample; (iii) not using a larger spectrum of music cadences. Future studies should link the kinetic and kinematic variables to the coordination and try to determine an optimal music cadence for older adults. The long-term effects in propulsive force according to different types of programs and exercises (e.g., walking, rocking, running, kicking, scissors, and jumping) should also be considered for further attempts.

## 5. Conclusions

Static and dynamic bilateral force production in older women induces different propulsive forces at various intensities. The cadence of 150 b·min^−1^ elicited higher bilateral propulsive forces for both exercises. Nevertheless, it seems that the static condition is the more suitable strategy to reduce asymmetries and to achieve a better coordination pattern in the elderly population.

## Figures and Tables

**Table 1:** Descriptive statistic (Mean ± SD) of propulsive peak force between the two water exercises and between the upper limbs at the same music cadence (*n* = 27).

**Cadences**	Variables	Static (HA)	Dynamic (RH)	*p*-Value	ES (*d*)
		Mean ± SD	Mean ± SD		
105 b·min^−1^	PF_D_ (N)	21.03 ± 4.25 **	20.41 ± 3.96	0.53	0.15
	PF_ND_ (N)	18.85 ± 4.24	18.62 ± 5.88	0.84	0.05
120 b·min^−1^	PF_D_ (N)	24.18 ± 4.40	22.25 ± 5.50 *	0.04	0.40
	PF_ND_ (N)	23.39 ± 3.89	19.69 ± 4.58	<0.01	0.89
135 b·min^−1^	PF_D_ (N)	28.59 ± 4.53 *	26.91 ± 5.56 **	0.15	0.34
	PF_ND_ (N)	26.74 ± 4.89	23.37 ± 6.17	<0.01	0.62
150 b·min^−1^	PF_D_ (N)	31.75 ± 5.55	28.34 ± 4.64 *	<0.01	0.68
	PF_ND_ (N)	30.35 ± 5.66	26.20 ± 5.80	<0.01	0.74

*, *p* ≤ 0.05 significant differences between PF_D_ and PF_ND_; **, *p* ≤ 0.01 highly significant differences between PF_D_ and PF_ND_; b·min^−1^, beats per minute; HA, horizontal adduction; *n*, number of subjects; N, Newton; PF_D_, propulsive peak force for dominant upper limb; PF_ND_, propulsive peak force for nondominant upper limb; RH, rocking horse.

**Table 2:** Descriptive statistic (Mean ± SD) for the symmetry index (SI) (*n* = 27).

Cadences	Variable	Static (HA)	Dynamic (RH)
		Mean ± SD	Mean ± SD
105 b·min^−1^	SI (%)	14.64 ± 10.75	22.08 ± 18.04
120 b·min^−1^	SI (%)	14.24 ± 9.55	18.86 ± 14.63
135 b·min^−1^	SI (%)	14.10 ± 13.79	18.53 ± 17.57
150 b·min^−1^	SI (%)	15.82 ± 13.37	16.18 ± 12.18

%, percentage; HA, horizontal adduction; *n*, number of subjects; RH, rocking horse; SI, symmetry index.

## Data Availability

The data presented in this study are available on request from the corresponding author.
